# Development and Validation of an Evaluation Questionnaire for the Healthy Early Life Moments in Singapore (HELMS) Program

**DOI:** 10.3390/jpm14090989

**Published:** 2024-09-17

**Authors:** Chee Wai Ku, Muhammad Ashraf Yusoff, Elvia Chin Boon Ng, Ruther Teo Zheng, Fabian Yap, Jerry Kok Yen Chan, See Ling Loy

**Affiliations:** 1 Department of Reproductive Medicine, KK Women’s and Children’s Hospital, 100 Bukit Timah Road, Singapore 229899, Singapore; jerrychan@duke-nus.edu.sg (J.K.Y.C.); loyseeling@duke-nus.edu.sg (S.L.L.); 2 Duke-NUS Medical School, 8 College Road, Singapore 169857, Singapore; fabian.yap.k.p@singhealth.com.sg; 3 Department of Obstetrics and Gynecology, KK Women’s and Children’s Hospital, 100 Bukit Timah Road, Singapore 229899, Singapore; mdashrafyusoff@gmail.com; 4 Yong Loo Lin School of Medicine, National University of Singapore, 10 Medical Drive, Singapore 117597, Singapore; elviang@u.nus.edu; 5 Endocrinology Service, Department of Pediatrics, KK Women’s and Children’s Hospital, 100 Bukit Timah Road, Singapore 229899, Singapore; zheng.ruther.teo@kkh.com.sg; 6 Lee Kong Chian School of Medicine, Nanyang Technological University, 59 Nanyang Drive, Experimental Medicine Building, Singapore 636921, Singapore

**Keywords:** preconception care, mobile health, metabolic health, evaluation questionnaire, implementation outcomes

## Abstract

Background/Objectives: Global fertility rates are declining due to metabolic and mental health challenges in women trying to conceive. The Healthy Early Life Moments in Singapore (HELMS) program aims to address these challenges through mobile health (mHealth)-enabled lifestyle interventions. However, the lack of validated evaluation tools for such programs makes it difficult to assess their feasibility and acceptability. To tackle this, a comprehensive evaluation questionnaire was developed and validated to determine if the HELMS preconception program’s implementation outcomes were achieved. Methods: The questionnaire development process included a literature review and a two-step validation process: content validation by five content experts and face validation by 20 HELMS participants. Content validation was assessed using the scale content validity index (S-CVI) based on relevance, clarity, simplicity, and ambiguity. Face validation with participants evaluated these criteria and the ease of completing the questionnaire. Internal consistency was assessed using Cronbach’s alpha among 49 participants. Results: The questionnaire achieved good S-CVI values for relevance (0.93), clarity (0.91), simplicity (0.94), and ambiguity (0.71). After expert feedback, the revised version scored highly among HELMS participants for relevance (100%), clarity (95%), simplicity (95%), and non-ambiguity (90%). A Cronbach’s alpha of 0.93 indicated good internal consistency. Conclusion: The HELMS evaluation questionnaire shows promise for evaluating similar mHealth-based lifestyle intervention programs globally.

## 1. Introduction

Today, a concerning trend is unfolding—a global decline in fertility rates, where fewer women are having children [1]. This decreasing trend can be attributed to various socioeconomic causes, including increased gender equality, rising childcare costs, and increased access to contraception and reproductive healthcare. Apart from evolving socioeconomic trends, poor preconception health is a significant cause of decreasing fertility rates [2]. The preconception period represents the most critical period where improvements in maternal health will not only reduce patients’ lifetime risks of metabolic diseases but also improve intergenerational health for the offspring [3]. Obesity and obesity-related metabolic conditions are becoming increasingly prevalent globally [4], which are associated with adverse maternal, pregnancy, and perinatal outcomes; long-term transgenerational effects on offspring with an increased risk of metabolic syndrome [5]; and higher risks of female subfertility [6]. Furthermore, in recent years, a worldwide increase in mental health challenges [7] may affect fertility rates. Feelings of distress can be associated with lower conception rates and poorer outcomes of assisted reproductive medicine. In addition, chronic stressors can also adversely affect ovarian health and reserves [8]. Despite the growing burden of metabolic disorders and mental health challenges globally and among preconception women affecting overall fertility rates, there are a lack of interventions to address this. Therefore, there is a pressing need to develop a comprehensive and integrated intervention to improve metabolic and mental health in women who are trying to conceive, thereby improving fertility and maternal-offspring outcomes.

The Healthy Early Life Moments in Singapore (HELMS) program, initiated by KK Women’s and Children’s Hospital in Singapore, aims to optimize maternal and child health and bridge this clinical gap [3]. The HELMS model of care integrates the preconception, pregnancy, and postpartum journey through the support, inform, guide, and nudge (SIGN) approach. The lifestyle intervention modules include the 6P model for nutrition and physical activity, mental health, and sleep, along with the 4S model of care that comprises screening, size, supplements, and sex [9]. This information is delivered through consultations with healthcare professionals, and digitally through a mobile health (mHealth) application, called e-HELMS. The success of lifestyle interventions, including the HELMS preconception program, hinges on adherence and ongoing engagement. Thus, as suggested by Proctor et al., ongoing evaluation of these programs through their implementation and service outcomes is key to ensuring their feasibility and acceptability to participants [10]. There is a paucity of validated questionnaires specifically designed to assess the implementation outcomes of lifestyle intervention programs, such as HELMS.

Existing validated questionnaires like the Acceptability of Intervention Measure (AIM), Intervention Appropriateness Measure (IAM), and Feasibility of Intervention Measure (FIM), primarily assess discrete implementation outcomes, whereas questionnaires such User Version of the Mobile Application Rating Scale (uMARS) and the mHealth App usability questionnaire (MAUQ) only focus on the usability of the mHealth delivery platforms [11,12,13]. These tools, while robust, do not fully capture the complexities of sophisticated lifestyle intervention programs like HELMS, as they often fail to address the nuances and interconnected nature of such interventions, thereby limiting their practical applicability. The AIM, IAM, and FIM [13] are leading indicators of implementation success, assessing the acceptability, appropriateness, and feasibility of interventions. However, these measures tend to assess specific outcomes in isolation and do not adequately reflect the contextual nuances of the program content. Therefore, these existing tools fall short of effectively evaluating the comprehensive implementation outcomes necessary for the HELMS program. Similarly, while the uMARS [11] and MAUQ [12] are tailored for mHealth applications, their focus remains on user interface and interaction rather than the holistic content within the program’s context. This gap underscores the need for developing a more integrated and contextually tailored evaluation tool that encompasses both the delivery mechanisms and the content of HELMS, ensuring a more thorough and effective assessment of its implementation outcomes.

Moreover, systematic reviews that scrutinize the effectiveness of existing evaluation tools for mHealth interventions further underscore these limitations [14,15]. These reviews reveal that while traditional evaluation tools have been effective in assessing basic elements such as usability, engagement, and app features, they largely neglect the complex, content-specific implementation outcomes critical to lifestyle interventions. This oversight is particularly significant given that the mHealth platform not only serves as a medium of information delivery but is also instrumental in the broader context of health interventions. Consequently, the need for an evaluation questionnaire that comprehensively addresses both the delivery mechanisms and the content specifics of the HELMS program becomes evident. Such a tool would enable a more nuanced understanding and assessment of the program’s effectiveness, ensuring that both the technological and substantive elements of the intervention are seamlessly integrated and effectively evaluated.

Therefore, in this study, we aim to develop and validate a novel questionnaire to assess the implementation outcomes of the preconception phase of the HELMS program. This validated questionnaire at the preconception phase will be a template for the evaluation of HELMS at the pregnancy and postpartum phases subsequently, and potentially serve as a reference for the evaluation of other lifestyle intervention programs. This initiative reflects our commitment to enhancing intervention effectiveness and adaptability to address the holistic needs of women in preconception health settings.

## 2. Materials and Methods

**Figure 1:**
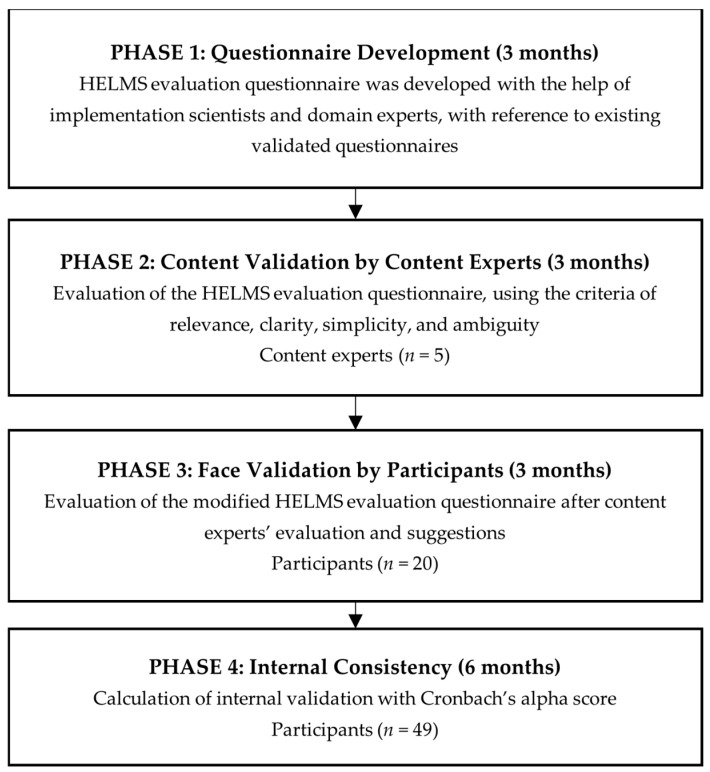
Flowchart of the validation process of the HELMS evaluation questionnaire.

The development and validation of the HELMS evaluation questionnaire comprised four phases: questionnaire development, content validation by content experts, face validation by participants, and internal consistency (Figure 1). Each of the first three phases (Phase 1–3) was conducted over a duration of 3 months, while Phase 4 was conducted over 6 months.

### 2.1. Phase 1: Questionnaire Development

The development of the HELMS evaluation questionnaire was grounded in a comprehensive review of available validated tools known for robustly measuring implementation outcomes. These included the AIM, IAM, and FIM [13], which provided foundational insights into critical dimensions of implementation science. Additionally, the uMARS [11] and the MAUQ [12] were referenced to integrate metrics specifically tailored to evaluate mHealth applications delivering lifestyle interventions. This integrative approach was directed by the framework proposed by Proctor et al. [10], focusing on six key implementation outcomes: acceptability, adoption, appropriateness, feasibility, effectiveness, and timeliness. The initial compilation resulted in a preliminary set of 14 questions (Appendix A). A Likert scale of 1 to 5 was employed to rate the level of agreement with the statements in the questionnaire (1 = “Strongly disagree”; 2 = “Disagree; 3 = “Neutral”; 4 = “Agree”; 5 = “Strongly agree”). The selection and distribution of these questions were strategically aligned with each domain’s perceived importance and complexity, as rigorously evaluated by a panel of experts in implementation science and mHealth. This tailored approach ensured that the questionnaire was not only comprehensive but also sensitive to the nuances of an mHealth-based lifestyle interventions such as the HELMS preconception program.

### 2.2. Phase 2: Content Validation by Content Experts

Five content experts were recruited to participate in the content validation stage of the HELMS evaluation questionnaire and provide their expert review. The backgrounds of the content experts are as follows: a pediatric emergency medicine clinician, a pediatric medicine clinician, an internal medicine clinician, and two implementation science researchers. All five content experts have experience in epidemiology and implementation science research. A minimum of five content experts has been shown to minimize bias due to chance agreement [16]. In the expert review form, content experts were asked to refer to the HELMS evaluation questionnaire and rate each of the individual 14 items on a four-point Likert scale, based on each of the four criteria: relevance, clarity, simplicity, and ambiguity [17]. A score of 1 is given if the expert rates the item a ‘3’ or ‘4’, and a score of 0 is given if the expert rates the item a ‘1’ or ‘2’ on a four-point Likert scale. All comments by content experts were reviewed. Modifications were then made to the HELMS evaluation questionnaire after the evaluation by content experts by the authors. The initial questionnaire and subsequent modifications after content expert evaluation are shown in Appendix A.

### 2.3. Phase 3: Face Validation by Participants

The modified HELMS evaluation questionnaire underwent an assessment of face validity by participants. Twenty HELMS participants in the preconception phase were recruited by random sampling to participate in the face validation phase of the HELMS evaluation questionnaire and completed a face validation survey for participants. The survey for participants consisted of seven questions, which is shown in Appendix B. Participants were asked to refer to the HELMS evaluation questionnaire and rate the overall questionnaire on a 5-point Likert scale [18].

### 2.4. Phase 4: Internal Consistency

The modified HELMS evaluation questionnaire was completed by 49 participants in the preconception phase (including those who participated in the face validation). These responses were used to calculate the internal consistency of the questionnaire using Cronbach’s alpha.

### 2.5. Statistical Analysis

In Phase 2 of the study, results from the expert review survey were used to calculate the content validity index (CVI) to ascertain the content validity of the HELMS evaluation questionnaire. The CVI is a widely used index calculating the level of consensus among a group of experts in evaluating a tool in relation to the content of study [19]. Individual item CVI (I-CVI) values for each of the four criteria of relevance, clarity, simplicity, and ambiguity can be calculated based on averaging the scores (0 or 1) for each item. Scale content validity index (S-CVI) is the CVI of all the questions and can be calculated by taking the average of all the I-CVI values. I-CVI and S-CVI values were calculated for the HELMS evaluation questionnaire for each of the abovementioned four criteria. The criterion value for S-CVI was set at 0.80 to be considered acceptable [20]. In Phase 3 of the study, the number of participants who responded with a “1” or “2” (negatively), “3” (neutral), and “4” or “5” (positively) for each question were obtained. The mean scores and standard deviation for each question were calculated. The internal consistency of the questionnaire was determined using the Cronbach’s alpha. A threshold of 0.70 and above is generally considered acceptable [21,22]. IBM SPSS Statistics version 29.0 was used to perform the above analyses.

## 3. Results

### 3.1. Content Validation by Content Experts

The I-CVI and S-CVI scores for each of the four criteria of the HELMS evaluation questionnaire are presented in Table 1. The S-CVI values for the criteria of relevance, clarity, simplicity, and ambiguity are 0.93, 0.91, 0.94, and 0.71 respectively.

The HELMS evaluation questionnaire subsequently underwent modifications based on these results. The phrasing of eight questions was modified and one question: “The HELMS preconception program is appealing to me” was removed to produce a 13-item HELMS evaluation questionnaire (Appendix A). This question was removed due to ambiguity of phrasing and redundancy with other questions.

### 3.2. Face Validation by Participants

Of the 20 women who participated in the face validation phase, 75% were Chinese (15/20), 20% were Malay (4/20), and 5% were Indian (1/20) (Table 2). Most had attained tertiary education (14/20, 70%), and were employed (20/20, 100%). The mean age was 34.1 ± 3.2 years, while the mean BMI was 27.5 ± 2.6 kg/m^2^. The responses of the abovementioned participants for the face validation are shown in Table 3. Most participants responded positively to the questionnaire, agreeing that the questions were relevant (100% of participants), clear (95%), simple and easy to understand (95%) and not ambiguous (90%). 95% of participants did not encounter any problems during the completion of the evaluation form.

Responses from 49 HELMS participants who went on to complete the questionnaire were used to determine Cronbach’s alpha. The Cronbach’s alpha for the final questionnaire consisting of 13 questions was 0.93, which indicated an acceptable level of the questionnaire’s internal consistency.

## 4. Discussion

The evaluation questionnaire designed to assess the implementation outcomes of the preconception phase of the HELMS program demonstrated acceptable content validity, face validity, and internal consistency. Content validity, face validity, and internal consistency are important to ensure that the questionnaire is reliable, representative of the program’s multifaceted aspects, and suitable for the target audience and their socio-cultural background [23,24]. This validated questionnaire at the preconception phase will serve as a template for the subsequent pregnancy and postpartum phases of HELMS.

Content validity of the questionnaire was achieved for the criteria of relevance, clarity, and simplicity as their S-CVI values met the minimum threshold of 0.80. However, for the criteria of ambiguity, the questionnaire achieved a S-CVI value of 0.71, which is under the threshold of acceptable S-CVI scores. The questionnaire consequently underwent modifications based on expert feedback. Subsequently, the final version of the questionnaire achieved high scores on face validity by participants, who agreed that the questionnaire was relevant, clear, simple to understand, and not ambiguous. Given the high positive scores from face validity and the fact that most participants (19/20, 95%) faced no difficulties in completing the questionnaire, no further amendments were made to the questionnaire.

We have meticulously adapted existing evaluation tools, including the AIM, IAM, FIM, uMARS, and MAUQ, [11,12,13] to better align with the specific requirements of the HELMS program. These adaptations, detailed in Appendix A, span various implementation domains and reflect a tailored approach to assessing our intervention. We did not merely adopt existing questions from these tools verbatim (questions 1–4, 6, 8–9); instead, each was carefully reworded and adjusted to more accurately address the unique challenges and contexts of the HELMS program. This included a rigorous content validation process involving domain experts who refined the questions to ensure they were contextually appropriate and technically precise. Additionally, recognizing gaps in the coverage of existing tools, we developed new questions specifically tailored for the HELMS program (questions 5, 7, 10–14). These questions are designed to measure implementation outcomes that were previously unaddressed by the standard tools, focusing on specific elements of the HELMS lifestyle intervention, such as participant engagement with the program content and the acceptability of the time commitments required. This innovative approach not only fills existing methodological gaps but also enhances the robustness and comprehensiveness of our evaluation framework, ensuring it effectively captures the nuanced dynamics of our intervention.

A major strength of the HELMS evaluation questionnaire, compared to existing questionnaires, is the length. Existing questionnaires such as the AIM, IAM, and FIM comprise brief four-item scales [13], while the uMARS [11] and MAUQ [12] consist of 20 and 21 items, respectively. Meanwhile, the validated HELMS evaluation questionnaire comprises 13 items, which is of intermediary length between the abovementioned questionnaires. The balance between questionnaire length and comprehensiveness is important, as participants tend to lose interest in longer questionnaires, rushing through or skipping questions, affecting the quality, reliability, and response rates [20]. There is no universally accepted optimal time for completing a questionnaire [25,26], though some studies suggest an ideal length of 13 min to obtain good response rates [27,28]. The estimated time taken to complete our questionnaire was 5–10 min. Compared to existing questionnaires, the HELMS evaluation questionnaire addresses implementation outcomes unique to the HELMS preconception program that is delivered on a mHealth platform. Taken together, the HELMS evaluation questionnaire has been well-received in the expert review and face validation stages.

With the successful validation of the HELMS evaluation questionnaire, it will be implemented subsequently to evaluate the HELMS preconception program. Implementation outcomes are crucial in the overall success of an intervention, as they directly impact its successful adoption, integration, and sustainability in real-world healthcare settings [10,29]. A systematic approach towards evaluating implementation outcomes aids in understanding the success factors of interventions and facilitating the development of tailored implementation strategies, thereby ensuring the efficient delivery of evidence-based practices [30]. The final HELMS evaluation questionnaire consists of 13 questions that serve to assess implementation outcomes of the overall program, such as acceptability, adoption, appropriateness, feasibility, effectiveness, and timeliness. These outcomes are congruent with the proposed taxonomy of implementation outcomes by Proctor et al. [10], which have the potential to identify contextual elements hindering implementation success. Additionally, participants may be selected for face-to-face interviews to explore the barriers and facilitators of the HELMS program. This qualitative study will complement the quantitative evaluation questionnaire to provide a holistic evaluation of the HELMS program and guide future implementation of similar lifestyle intervention programs like HELMS.

Beyond the HELMS preconception program, the HELMS evaluation questionnaire may be extrapolated to other similar lifestyle interventions delivered on a mHealth platform. Although specific aspects may differ based on different target populations and/or health goals, several common principles underpin successful lifestyle intervention programs. These principles include, but are not limited to, individualization, behavioral modification, multi-component approaches, goal setting, and social support [31,32,33,34]—all of which are characteristics of HELMS. Furthermore, the HELMS evaluation questionnaire has been developed and validated in the multi-ethnic Asian context of Singapore. The pre-existing questionnaires previously discussed were mainly validated and implemented in Western contexts, namely Australia [11] or the United States of America [12,13]. The phrasing and structure of various components of the HELMS evaluation questionnaire may therefore be more acceptable and understandable to Asian individuals, allowing for potentially greater seamless extrapolation into other similar lifestyle intervention programs.

### Strengths and Limitations

The newly developed and validated HELMS evaluation questionnaire is the first of its kind to assess implementation and usability outcomes for the HELMS preconception program in a single questionnaire. Unlike existing platform-specific mHealth questionnaires, the HELMS evaluation questionnaire consists of questions specific to the implementation outcomes of the HELMS program. A limitation of this study is that only one round of content validation with content experts was carried out. However, the robust face validation results, which reflect direct feedback from the HELMS program participants, reinforce our confidence in the revisions made and the overall quality of the questionnaire. Nevertheless, it is prudent to consider additional rounds of content validation in future iterations of the questionnaire to further enhance its reliability and applicability. However, the questionnaire achieved generally good CVI values in the first round of content validation, and subsequently only underwent minor changes in phrasing and the removal of one question. Face validation by participants also yielded good results on the questionnaire. Another potential limitation would be that the face validation phase was conducted purely quantitatively. While this approach was effective in assessing specific criteria (relevance, clarity, simplicity, and ambiguity), it did not capture qualitative feedback that could provide deeper insights into the user experience of the HELMS evaluation questionnaire. Future studies should be planned to incorporate qualitative methods to gather comprehensive feedback, enhancing understanding of the questionnaire’s practical impact and user satisfaction.

A potential source of bias in this study is that participants assessing the face validity of the HELMS evaluation questionnaire are existing HELMS preconception program participants. Hence, they may have an existing impression of the HELMS preconception program, which may positively or negatively influence their perception of the validity of the HELMS evaluation questionnaire and give rise to biased responses. However, notably, the HELMS evaluation questionnaire is meant to be administered to participants who have undergone the entire HELMS program. Therefore, engaging the HELMS participants in the face validation phase may be beneficial as they would be able to evaluate the evaluation questionnaire in the specific context of HELMS and provide relevant feedback.

## 5. Conclusions

The results of this study demonstrated that the HELMS evaluation questionnaire has acceptable levels of content validity, face validity, and internal consistency. Therefore, the HELMS evaluation questionnaire will be administered to HELMS participants to comprehensively evaluate the HELMS preconception program. Subsequently, there is great potential for this questionnaire to be adopted for the systematic evaluation of similar mHealth-based comprehensive lifestyle intervention programs globally.

## Figures and Tables

**Table 1:** Content validation of the HELMS Evaluation questionnaire by content experts.

Item	Relevance	Clarity	Simplicity	Ambiguity
I-CVI ^a^				
Q1	0.80	0.80	0.80	0.80
Q2	0.80	0.40	0.80	0.40
Q3	0.80	1.00	0.80	0.60
Q4	0.80	0.80	0.80	0.60
Q5	0.80	0.80	1.00	0.40
Q6	1.00	1.00	1.00	0.80
Q7	1.00	1.00	1.00	0.80
Q8	1.00	1.00	1.00	0.80
Q9	1.00	1.00	1.00	0.80
Q10	1.00	1.00	1.00	0.80
Q11	1.00	1.00	1.00	0.80
Q12	1.00	1.00	1.00	0.80
Q13	1.00	1.00	1.00	0.80
Q14	1.00	1.00	1.00	0.80
S-CVI ^b^	0.93	0.91	0.94	0.71

^a^ I-CVI: Individual Item Content Validity Index; ^b^ S-CVI: Scale Content Validity Index.

**Table 2:** Demographics of the HELMS participants involved in the face validation phase.

Demographic	Total (*n* = 20)
Age (years)	34.1 ± 3.2
Body mass index (kg/m^2^)	27.5 ± 2.6
Ethnicity, *n* (%)	
Chinese	15 (75.0)
Malay	4 (20.0)
Indian	1 (5.0)
Highest education, *n* (%)	
Below tertiary	6 (30.0)
Tertiary and above	14 (70.0)
Employment status, *n* (%)	
Unemployed	0 (0.0)
Employed	20 (100.0)
Parity, *n* (%)	
0	10 (50.0)
1 or 2	10 (50.0)

**Table 3:** Face validation of the HELMS evaluation questionnaire by HELMS participants.

Survey Question	Number of Participants Who Responded with a Score of 1 or 2 (*n*)	Number of Participants Who Responded with a Score of 3 (*n*)	Number of Participants Who Responded with a Score of 4 or 5 (*n*)	Mean Score
Q1. The questions in the evaluation form are relevant	0	0	20	4.60 ± 0.50
Q2. The questions in the evaluation form are clear	0	1	19	4.35 ± 0.59
Q3. The questions in the evaluation form are simple and easy to understand	0	1	19	4.55 ± 0.60
Q4. The questions in the evaluation form are not ambiguous	1	1	18	4.40 ± 0.99
Q5. I did not encounter any problems during the completion of the evaluation form	1	0	19	4.50 ± 0.76

## Data Availability

The data presented in this study are available on request from the corresponding author due to patient confidentiality and privacy reasons.

## Appendix A

**Table A1:** Initial HELMS questionnaire and subsequent modifications after content expert validation.

Initial Questionnaire	Questionnaire after Content Expert Evaluation	Domain	Modified from Existing Questionnaires
How would you rate your *overall satisfaction* with the HELMS preconception program?	I am *satisfied* with the HELMS preconception program.	Acceptability	MAUQ
2. The HELMS preconception program *meets my approval*.	The HELMS preconception program *meets my expectations*.	Acceptability	AIM
3. The HELMS preconception program is *appealing* to me.	*Removed*.	Acceptability	AIM
4. I *like* the HELMS preconception program.	I *like* the HELMS preconception program.	Acceptability	AIM
5. How often do you *apply the knowledge learnt* from the program in your *daily life*?	How often do you *apply the knowledge learnt* from the program in your *daily life*?	Adoption	*New question*
6. How *useful* do you think is the *preconception information* and *education* provided to you?	How *useful* is the *preconception information* and *education* provided to you?	Appropriateness	MAUQ
7. Is the knowledge gained through the HELMS preconception program *relevant* to you?	Is the knowledge gained through the HELMS preconception program *relevant* to you?	Appropriateness	*New question*
8. The HELMS preconception program is *suitable* for me.	The HELMS preconception program is *suitable* for me.	Appropriateness	IAM
9. The HELMS preconception program is *easy to follow*.	The HELMS preconception program is *easy to understand*.	Feasibility	FIM, uMARS
10. How *motivated* are you to make changes to your lifestyle after joining HELMS?	How *motivated* are you to make changes to your lifestyle after joining HELMS?	Effectiveness	*New question*
11. Are you now more *confident* in your knowledge of healthy lifestyle after joining HELMS?	How *confident* are you in applying your knowledge gained of healthy lifestyle after joining HELMS?	Effectiveness	*New question*
12. Was the amount of *time spent* during the HELMS *preconception visits* (baseline, 6-month follow-up, and 12-month exit visit, if applicable) acceptable (excluding time spent on questionnaires)?	Was the amount of *time spent* during the HELMS *preconception in-person visits* acceptable (excluding time spent on questionnaires)?	Timeliness	*New question*
13. Was the amount of *time spent* during the HELMS *preconception teleconsultation* (3-month and 9-month follow-up, if applicable) acceptable?	Was the amount of *time spent* during the HELMS *preconception teleconsultation* acceptable?	Timeliness	*New question*
14. The *number of visits* (both in-person and teleconsultation) during the HELMS preconception phase is appropriate.	The *number of visits* (both in-person and teleconsultation) during the HELMS preconception phase was just right.	Timeliness	*New question*

## Appendix B

**Table A2:** Face validation survey questions.

Questions	Answer Choices
Q1. The questions in the evaluation form are relevant.	5-point Likert scale
Q2. The questions in the evaluation form are clear.	
Q3. The questions in the evaluation form are simple and easy to understand.	
Q4. The questions in the evaluation form are not ambiguous.	
Q5. I did not encounter any problems during the completion of the evaluation form.	
Q6. Is there anything that can be done to improve the evaluation form?	“Yes”, “No”, or “Not sure”
Q7. If yes to Qn 6, can you please share with us what can be done to improve the evaluation form?	Open-ended
